# Volumetric and Asymmetric Index Analysis of Subcortical Structures in Multiple Sclerosis Patients: A Retrospective Study Using volBrain Software

**DOI:** 10.7759/cureus.55799

**Published:** 2024-03-08

**Authors:** Ayla Tekin, Buket Rende, Hüsnü Efendi, Sena Destan Bunul, Özgür Çakır, Tuncay Çolak, Sibel Balcı

**Affiliations:** 1 Anatomy, Kocaeli University, Kocaeli, TUR; 2 Anatomy, European Vocational School, Kocaeli Health and Technology University, Kocaeli, TUR; 3 Neurology, Kocaeli University, Kocaeli, TUR; 4 Radiology, Kocaeli University, Kocaeli, TUR; 5 Anatomy, Faculty of Medicine, Kocaeli University, Kocaeli, TUR; 6 Biostatistics and Medical Informatics, Kocaeli University, Kocaeli, TUR

**Keywords:** magnetic resonance imaging, s: multiple sclerosis, subcortical, volume, bilateral asymmetry

## Abstract

Introduction

Multiple sclerosis (MS) is a chronic and autoimmune disease that has a significant influence on the central nervous system, such as the brain and spinal cord, affecting millions of individuals globally. Understanding the connection between subcortical brain regions and MS is crucial for effective diagnostic and therapeutic approaches for treating this disabling disease. This study explores the relationship between volume and contours of asymmetry index of subcortical brain regions in individuals with MS using volBrain software (https://www.volbrain.net; developed by José V. Manjón (Valencia Polytechnic University, Valencia, Spain) and Pierrick Coupé (University of Bordeaux, Bordeaux, France)).

Methods

In our retrospective investigation, we admitted 100 Turkish individuals, comprising 50 patients diagnosed with relapsing-remitting MS (RRMS) (24 (48%) males and 26 (52%) females) and 50 healthy controls (23 (46%) males and 27 (54%) females), registered between October 2017 and February 2022 for five years and underwent assessment in the radiology department at the Teaching and Research Hospital of Kocaeli University; 1,150 Turkish patients were excluded from our study based on our exclusion criteria. We used magnetic resonance imaging with a 3-Tesla (3T) scanner and volBrain software to assess volumes (cm^3^) and asymmetry indexes due to asymmetry for different levels of atrophy of total intracranial, total brain, gray matter, white matter, and subcortical regions, the most affected regions in MS patients for both patient and control cohorts.

Results

Statistical analysis revealed a significant difference between patient and control groups (p < 0.001), with patient group mean age at 38.32 years and control group mean age at 32.88 years. Patient group exhibited lower values for total intracranial, total brain, gray matter, white matter, and cerebrospinal fluid volume compared to control group (p < 0.05). The results indicated a statistically significant decrease (p < 0.05) in the values for total intracranial and total brain volume, whereas all other values remained unchanged. We compared volumes of subcortical structures on the right and left sides and found that the putamen, thalamus, and globus pallidus had statistically lower values in the patient group than in the control group (p < 0.001), apart from the lateral ventricle. Furthermore, our retrospective investigation demonstrated a statistically significant difference in the globus pallidus asymmetry index, indicating a preference for the patient group (p < 0.05). A lower asymmetry index value signifies a larger volume for the right side of the subcortical regions of the brain when compared to the left side.

Conclusion

Brain atrophy, although characterized by irreversible tissue damage, is targeted by therapeutic interventions to prevent progression. It is, therefore, imperative to develop a universally accepted measurement standard for subcortical structures that also considers the inherent variability present within each structure. Our findings serve as an important basis and indicator for the determination of subcortical atrophy and asymmetry in MS, the prognosis of the disease, and the etiology of clinical symptoms. Subsequent research may benefit by adopting the novel approach of considering brain atrophy as an outcome rather than a predictor, thereby facilitating the elucidation of the intricate biological mechanisms that give rise to volume loss.

## Introduction

Multiple sclerosis (MS) is the most common neurological disorder caused by the immune system’s attacks and inflammatory mechanisms on the structures of the central nervous system, especially the brain and spinal cord [1]. In particular, inflammatory damage presents in the myelin sheath, which protects nerve fibers called the white matter, and this situation leads to the generation of MS [1]. This inflammatory damage can affect the neuronal communication between cells and trigger a variety of symptoms [1]. The autoimmunity present in MS leads the immune system to attack cells and tissues by perceiving them as foreign material. This is a significant factor in the progression of MS, as it targets the nerve fibers and induces damage to the myelin sheath, leading to communication impairments among nerve cells [1]. The most common symptoms of MS are vision malfunction, muscle atrophy, exhaustion, disequilibrium, and sensory and coordination impairments [1]. The condition has the potential to deteriorate with time and may significantly impair an individual’s overall well-being [2]. The etiology of MS is not yet fully understood, but it is generally considered to be the result of a complex interplay between genetic background, environmental factors, and immunological dysfunction that could activate autoimmunity [2]. MS is typically diagnosed through a combination of clinical assessment, MRI scans, and tests associated with the cerebrospinal fluid [3]. The treatment of MS primarily aims to control the symptoms and decelerate the progression of the illness [4]. Specifically, the administration of drugs, physical therapy, rehabilitation, and implementation of lifestyle changes are used to control the disease [4].

MRI is an indispensable diagnostic and management tool for MS [3]. MRI is a commonly preferred imaging technique that aids in the diagnosis and monitoring of various diseases in medicine [1]. MRI is used to evaluate the brain, spinal cord, joints, organs, muscles, and other tissues [3,5]. MRI can identify lesions in the brain and spinal cord and reveal their location and severity, which is important for effective treatment of MS [3,6]. MRI is essential to assess the state of the disease and devise effective treatment strategies, thereby facilitating appropriate management of MS [5]. Additionally, MRI is highly valuable for tracing modifications in volume over time and pinpointing the connections between these changes and the disease progression [6]. MRI is also significant in providing insights into the pathology and physiology of MS [7]. MRI also generates the data necessary to perform subcortical morphometry. Subcortical morphometry is the preferred method to investigate the size and shape of subcortical structures in brain imaging and neurological research in an analytical manner [8]. This method processes MRI data to quantify the density and volume of specific areas of the brain, offering critical insights for the diagnosis, progression, treatment of neurological disorders, and morphological alterations including degeneration, hypertrophy and persistence and the specific morphological changes in this region displayed neurodegenerative disorders [9,10]. Subcortical morphometry is often used by doctors and scientists to research neurological diseases such as Alzheimer’s disease, Parkinson’s disease, schizophrenia, and MS [8,9].

Many software programs, including volBrain (https://www.volbrain.net; developed by José V. Manjón (Valencia Polytechnic University, Valencia, Spain) and Pierrick Coupé (University of Bordeaux, Bordeaux, France)), use MRI to measure brain volume more accurately. Through this, it is possible to detect any irregularities [11]. Many automated and semi-automated programs calculate the brain volume by performing three-dimensional (3D) brain MRI with 1-mm-thick slices [11]. volBrain, a web-based software tool and platform for automatic image segmentation, can be used for assessing brain MRI images [11].volBrain can automatically measure intracranial space, white matter, gray matter, cerebrospinal, cerebellum, cerebrum, brainstem, lateral ventricle volumes, and subcortical gray matter [11]. volBrain offers automated image processing and provides precise volumetric measurements of various subcortical structures within the brain [11]. The information obtained from volBrain is useful for both diagnosing and monitoring brain diseases such as MS. volBrain employs normative data to assess any disparities between an individual’s brain structure measurements and those of a healthy population.

MRI images of individuals with MS typically reveal atrophy in the volume of subcortical regions and asymmetry [12]. These findings are related to the weakening symptoms of the disease [13]. Our study explored the relationship between the volume and asymmetry index of the subcortical structures in patients with MS. In addition, we measured the volumes of subcortical structures in brain MRI images of relapsing-remitting MS (RRMS) patients using volBrain software and compared these with the volume values in MRI images of control subjects within the same age range. The development of a universally accepted measurement standard for subcortical structures is imperative, and it is certainly important to recognize and take into consideration the inherent variability present within each structure. Furthermore, we provide a volumetric dataset for tracking MS patients as a preliminary basis for subsequent investigations.

## Materials and methods

Participants

In this retrospective study, we assessed 100 Turkish individuals, consisting of 50 patients diagnosed with RRMS (24 (48%) males and 26 (52%) females) and 50 healthy controls (23 (46%) males and 27 (54%) females), who were recorded in the hospital registry system between October 2017 and February 2022 for five years and attended the radiology department at the Teaching and Research Hospital of Kocaeli University. All participants underwent a 3-Tesla (3T) MRI and an Expanded Disability Status Scale (EDSS) test. The hospital collected the clinical and demographic data of the patients. In this study, we measured and compared the total intracranial, brain, gray matter, white matter, and subcortical area volumes and asymmetries of the patients and controls.

Inclusion and exclusion criteria

In the present study, we enrolled healthy individuals and patients diagnosed with RRMS who had undergone 3T MRI with an age range of 20-40 years among participants and shared comparable sex and age distributions. Patients with MS had a disease duration between zero and five years and an EDSS score between zero and 1.5 years. The criteria for exclusion from our study comprised neoplastic, degenerative, or vascular pathologies that might be mistaken for MS, the presence of any cranial pathology, including demyelinating findings, in the control group, and inadequate MRI sequences. A total of 1,200 Turkish patients were examined retrospectively for our investigation. We excluded 1,150 patients with an EDSS score of more than 1.5 and a disease duration of more than five years who did not have a 3T-MRI image and had our predefined exclusion criteria. In our study, the gender factor was not determinative in terms of inclusion or exclusion criteria.

Ethical approval

Our research was approved by the Kocaeli University Non-Interventional Clinical Research Ethics Committee, with the decision dated July 13, 2023, and numbered KOÜ-GOKAEK-2023/12.01.

Structural MRI protocol

All participants, including both patients and healthy controls, underwent cranial MRI using a 3T scanner equipped with an eight-channel head coil. The MRI protocol consisted of an axial 3D T1 turbo field echo sequence with the following specifications: TR = 11 msec; TE = 5 msec; slice thickness = 1 mm; slice gap = 0 mm; and fluid-attenuated inversion recovery with parameters TR = 11,000 msec, TE = 140 msec, slice thickness = 5 mm, slice gap = 0.5 mm, and matrix size = 512 × 512.

Quantitative image analyses

We transferred the 3D T1-weighted brain MRI data of the 100 participants to a personal computer using a picture archiving and communication system. The following steps were taken to process the routine 3D T1 MRI of both the patient and control groups and exported and saved Digital Imaging and Communications in Medicine (DICOM)-format images in a separate folder on the computer. We opened the dcm2niigui file using MRIcron software (www.nitrc.org; developed by Chris Rorden, McCausland Center for Brain Imaging, University of South Carolina, Columbia, SC) and dragged the folder containing the DICOM images to the dcm2nii application. We set the output format to compressed Functional Magnetic Resonance Imaging of the Brain (FMRIB) Software Library (FSL) four-dimensional Neuroimaging Informatics Technology Initiative (NIfTI, .nii). The dcm2nii software converted the DICOM images to NIfTI format, which was utilized by the brain imaging tool. We then converted the NIfTI file into a WinRAR file and added it to the archive.

**Figure 1:**
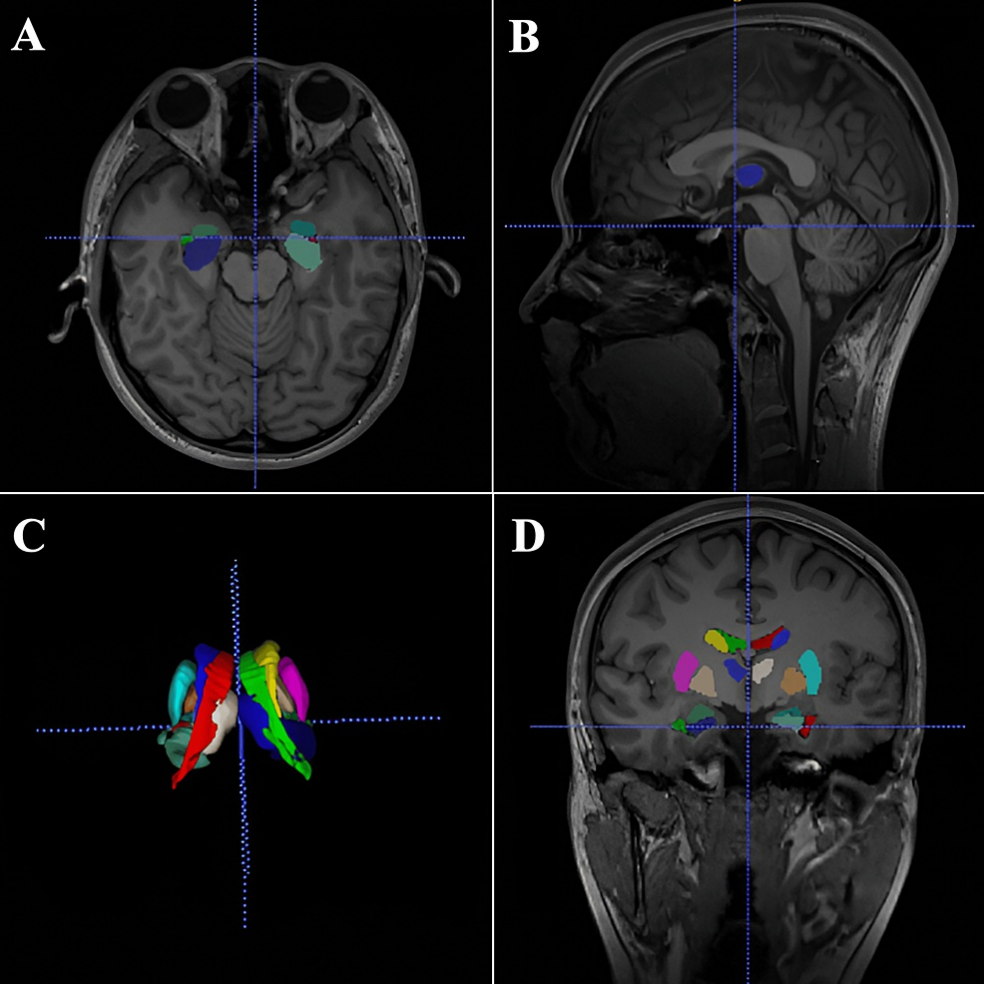
Colorization and three-dimensional modeling of volBrain data using ITK-SNAP (scale bar, 1 cm × 10 scale) in the (A) transverse plane, (B) sagittal plane, (C) subcortical structures, and (D) coronal plane.

Subsequently, we accessed volBrain via a search engine, and we chose a NIfTI file from the file section of the archive. The volume measurement results for the individual were promptly emailed to the user’s designated email address in the form of a portable document format (PDF) file upon provision of age and gender information [11]. We converted the volBrain data into 3D images by using Insight Segmentation and Registration Toolkit - Simple Nucleus Analysis Programme (ITK-SNAP; developed by student teams led by Guido Gerig, NYU Tanden School of Engineering, Brooklyn, NY) (http://www.itksnap.org) and MRIcroGL (https://www.nitrc.org/projects/mricrogl; developed by Chris Rorden, McCausland Center for Brain Imaging, University of South Carolina, Columbia, SC) (Figure 1). In addition, we calculated the asymmetry index by using the (L-R)/[(L+R)/2]×100 formula [14].

Statistical analysis

Statistical analysis was performed in a formal manner using IBM SPSS 20.0 (IBM Corp., Armonk, NY). We evaluated the normal distribution of the data using the Kolmogorov-Smirnov and Shapiro-Wilk tests. We reported normally distributed variables as mean ± SD, while we reported non-normally distributed variables as median (25th-75th percentiles). Categorical variables were presented as frequencies (percentages). We determined the differences between groups using independent sample t tests and Mann-Whitney U tests, while we identified the relationships between categorical variables using chi-square analysis. In hypothesis testing, a p value of less than 0.05 was considered statistically significant.

## Results

A total of 100 Turkish individuals participated in this study, including 50 patients diagnosed with RRMS (24 (48%) males and 26 (52%) females) and 50 healthy controls (23 (46%) males and 27 (54%) females). The difference in average age between the patient and control groups was statistically significant (p < 0.001), with the patient group having an average age of 38.32 years and the control group having an average age of 32.88 years. Additionally, there was a decrease in white matter, gray matter, total brain, total intracranial, and cerebrospinal fluid volume in the patient group compared to the control group; however, only the values for total brain and total intracranial volume showed a statistically significant decrease (p < 0.05), as shown in Table 1. 

**Table 1:** Comparison of age and volumes of total brain regions between the total, control, and patient groups. The parts in bold are statistically significant (p < 0.05). M, median; Q1-Q3, 25th to 75th percentiles; SD, standard deviation. *Independent sample t test; **Mann-Whitney U test.

Variables	Total	Control	Patient	t value	p value
Age (years), mean ± SD	35.06 ± 8.2	32.88 ± 6.87	38.32 ± 8.74	3.457	0.001*
White matter (cm^3^), mean ± SD	546.41 ± 192.65	559.10 ± 141.94	533.71 ± 233.48	-0.657	0.513*
Gray matter (cm^3^), mean ± SD	603.06 ± 195.37	633.70 ± 177	572.43 ± 209.46	-1.580	0.117*
Total brain (cm^3^), mean ± SD	1,149.47 ± 199.50	1,192.81 ± 175.89	1,106.14 ± 213.63	-2.215	0.029*
Total intracranial (cm^3^), M (Q1-Q3)	1,326.34 (1,230.46-1,430.10)	1,344.63 (1,273.83-1,456.82)	1,290.34 (1,222.26-1,410.03)	-	0.047**
Cerebrospinal fluid (cm^3^), M (Q1-Q3)	162.49 (117.15-231.64)	157.33 (116.11-305.79)	165.27 (131.16-214.03)	-	0.504**

In this study, the volumes of the subcortical structures on both sides of the brain were compared between the patient and control groups. The results showed that the putamen, thalamus, and globus pallidus had significantly lower volumes in the patient group than in the control group (p < 0.001), except for the lateral ventricle. The volumes of the right and left caudate, hippocampus, amygdala, and accumbens were lower in the patient group; however, the difference was not statistically significant (p > 0.05), as shown in Table 2.

**Table 2:** Comparison of volumes of subcortical areas in right and left sides of the brain between the control and patient groups. The parts in bold are statistically significant (p < 0.05). M, median; Q1-Q3, 25th to 75th percentiles; SD, standard deviation. *Independent sample t test; **Mann-Whitney U test.

Subcortical structures	Control	Right				Left		
		Patient	t value	p value	Control	Patient	t value	p value
Lateral ventricles, M (Q1-Q3)	0.88 (0.03-4.80)	3.54 (1.79-7.36)	-	0.001**	0.96 (0.01-5.83)	4.15 (1.43-8.98)	-	0.001**
Caudate, M (Q1-Q3)	4.28 (3.54-5.22)	3.62 (2.91-5.01)	-	0.079**	3.77 (2.97-5.12)	3.43 (2.46-4.92)	-	0.181**
Putamen, mean ± SD	4.56 ± 1.70	3.05 ± 1.29	-5.016	<0.001*	4.74 ± 1.53	3.10 ± 1.23	-5.892	<0.001*
Thalamus, mean ± SD	6.59 ± 2.15	4.99 ± 1.59	-4.350	<0.001*	6.42 ± 2.07	4.85 ± 1.48	-4.220	<0.001*
Globus pallidus, M (Q1-Q3)	1.06 (0.66-1.32)	0.46 (0.27-0.90)	-	<0.001**	1.00 (0.72-1.35)	0.39 (0.18-0.93)	-	<0.001**
Hippocampus M (Q1-Q3)	2.22 (1.26-3.13)	1.69 (1.11-3.14)	-	0.308**	2.21 (1.14-3.54)	2.14 (1.45-3.53)	-	0.471**
Amygdala, M (Q1-Q3)	0.12 (0.03-0.39)	0.04 (0.00-0.48)	-	0.250**	0.10 (0.03-0.43)	0.02 (0.00-0.39)	-	0.057**
Accumbens, M (Q1-Q3)	0.06 (0.01-0.18)	0.04 (0.00-0.17)	-	0.553**	0.05 (0.00-0.14)	0.02 (0.00-0.23)	-	0.831**

After comparing the asymmetry indexes of the subcortical area structures between the patient and control groups, we observed that there were statistically significant differences in favor of the patient group for the asymmetry in the globus pallidus (p < 0.05). The asymmetry index values in the subcortical area, excluding the globus pallidus, were also higher in the patient group; however, the difference was not statistically significant (p > 0.05), as shown in Table 3.

**Table 3:** Comparison of the volume of asymmetry indexes of subcortical areas on the right and left sides of the brain between the control and patient groups. The parts in bold are statistically significant (p < 0.05). M, median; Q1-Q3, 25th to 75th percentiles; SD, standard deviation. *Independent sample t test; **Mann-Whitney U test.

Index of subcortical structures	Control	Patient	t value	p value
Caudate asymmetry index, M (Q1-Q3)	3.96 (-2.71-15.98)	4.57 (-5.15-16.48)	-	0.831**
Putamen asymmetry index, M (Q1-Q3)	-2.69 (-12.78-3.52)	-3.89 (-10.84-6.11)	-	0.777**
Thalamus asymmetry index, mean ± SD	-1.54 ± 15.89	-2.62 ± 10.05	0.406	0.686*
Globus pallidus asymmetry index, M (Q1-Q3)	-1.16 (-10.92-11.55)	-10.52 (-31.52-2.41)	-	0.016**
Hippocampus asymmetry index, M (Q1-Q3)	-2.69 (-21.32-12.66)	2.75 (-28.83-15.67)	-	0.981**
Amygdala asymmetry index, mean ± SD	-1.76 ± 112.82	24.56 ± 117.61	-1.142	0.256*
Accumbens asymmetry index, mean ± SD	-0.26 ± 114.47	-2.21 ± 128.80	0.080	0.936*

## Discussion

In this retrospective study, we compared the volume and asymmetry of subcortical structures in RRMS patients and healthy controls. We found that RRMS patients had greater volume loss and more significant asymmetry in subcortical structures due to atrophy compared to healthy controls. The findings of our research emphasize the potential utility of measurement of the volume and asymmetry of subcortical structures as a biomarker of disease progression and changes in clinical symptoms in MS. Previous research on MS has mainly focused on assessing the volumes of the entire brain and subcortical regions as indicators of clinical decline. It has been widely acknowledged that MS patients suffer from significant demyelination and axonal damage, leading to substantial loss of both regional and overall brain volumes [15]. The rate of brain atrophy in MS patients is greater than that observed in healthy aging individuals [16]. Therefore, neuroradiological imaging plays a crucial role in assessing patients with MS. MRI is a reliable method for measuring brain atrophy and evaluating disease progression in MS [17]. Gray matter atrophy and asymmetry are commonly observed in MS and provide valuable insights into the etiology, progression, and cognitive decline associated with the disease [18]. Asymmetry is a common finding on brain MRI in patients with MS and is linked to the symptoms of the disease [19]. As subcortical structures in the brain do not experience atrophy evenly on both sides, asymmetry in these structures can be considered an early indicator of regional atrophy [20]. Usually, negative values of the asymmetry index represent a larger volume on the right side than on the left side.

Furthermore, MS is a neurological disorder that is associated with substantial volume loss in both gray and white matter as the disease progresses, which has a direct impact on the clinical course of the patient. This condition leads to a significant reduction in the volume of the brain, which has been widely documented in studies [21]. Eshaghi et al. found that the average age of the participants in their study was 38.32 ± 8.74 and 32.88 ± 6.87 for the patients and controls, respectively, and this difference was significant statistically [18]. Similarly, Ramezani et al. conducted a study on patients with RRMS and found that the average age of the patients was 34.22 ± 7.23, and the average age of the control group was 41.44 ± 8.06 [19]. Given that MS is most commonly diagnosed in the age range of 20-40 years, the mean age of MS patients in our study (38.32 ± 8.74) was consistent with the literature in terms of the etiology of the disease.

Atrophy of subcortical structures

The demyelination of neurons in MS often results in cytotoxicity and subsequent atrophy of various tissues [22]. In our research, we demonstrated that the patient group exhibited lower values for total brain, total intracranial, white matter, gray matter, and cerebrospinal fluid volume than the controls. Specifically, the total brain and intracranial volumes were significantly lower, as shown in Table 1. In contrast to our findings, a study by Ramezani et al. revealed no significant disparities in overall intracranial volume and gray matter volume between the control group and patients with RRMS, whereas white matter volume was found to be statistically lower in the patient group [19]. These findings suggest that a loss of volume in different areas may occur in MS, even in the early stages.

The mechanisms that contribute to the damage sustained by the gray matter in MS are distinct from those that impact the white matter and are thought to arise from a confluence of factors, including axon loss resulting from inflammation-induced degeneration, followed by Wallerian degeneration and post-inflammatory neurodegeneration, potentially stemming from a failure of remyelination. However, it is also notable that some observed shrinkage is not directly linked to focal lesions [23-25]. Volume loss or atrophy of subcortical structures in MS is clinically important for measurement and monitoring using MRI, and the mechanism of the loss of these structures remains to be fully elucidated [24]. Atrophy can occur in all subcortical structures but is more common in the thalamus, caudate, and hypothalamus [25]. In MS, volume loss in the subcortical region occurs early and precedes measurable whole-brain volume loss [18]. In our study, we found that the putamen, thalamus, and globus pallidus had significantly lower volumes in the patient group than in the control group, except for the lateral ventricle, as shown in Table 2. The right and left caudate, hippocampus, amygdala, and accumbens volumes were also lower in patients but were not statistically significant. Our finding suggests that subcortical structures are atrophied in this patient group, and this situation may indicate the prognosis of the disease. In this study, which included patients with MS disease duration between zero and five years and EDSS between zero and 1.5 years, atrophy was observed in subcortical structures even in the early period of the disease compared to the controls, which is in accordance with previous studies.

Asymmetry of subcortical structures

In a prior study, Magon et al. found a correlation between alterations in the thalamus’ structure and function in individuals with MS and their level of disability, as well as fatigue, behavioral changes, and cognitive dysfunction [26]. Furthermore, another study measuring thalamic volumes and calculating thalamic asymmetry indexes found that MS patients have lower overall thalamic volume compared to healthy controls, leading the authors to suggest that reduced thalamic volume indicates clinical deterioration [19]. Atrophy of the putamen and globus pallidus may negatively affect movement regulation, motor function, coordination, and cognitive abilities in patients with MS. In another study, Trufanov et al. found a notable difference in the volume of the pallidum and left nucleus accumbens between a control group and RRMS patients [27]. The volumes of these structures in the control group were notably greater than those in RRMS patients [27]. Additionally, the authors established a correlation between fatigue in a mixed group of patients and the volumes of the caudate nucleus, putamen, thalamus, and accessory nucleus [28]. Our study’s findings indicated that, unlike other subcortical structures, the lateral ventricle did not tend to decrease in volume in MS patients, which was associated with inflammation and was consistent with the literature [29]. In literature, a research showed that several key areas of the brain, including the hippocampus, amygdala, and nucleus accumbens, which had critical roles in memory, mood regulation, and emotional responses, were extensively affected by MS. These structures have been shown to experience significant demyelination, neuronal loss, and synaptic loss, but no statistically significant differences were observed in our study. The volume loss in these areas has been linked to cognitive impairment [30].

The detection of asymmetry in specific brain structures, including the globus pallidus, may act as an initial indicator of regional brain atrophy [20]. In this study, we analyzed the asymmetry indexes in subcortical structures and found that the global pallidus was the sole brain region that exhibited a statistically significant difference between the patient and control groups, as shown in Table 3. The subcortical area, excluding the globus pallidus, showed higher asymmetry index values in the patient group, but the difference was not statistically significant, as shown in Table 3. Globus pallidus asymmetry links to motor dysfunction in MS, caused by atrophy in the right and left hemispheres, leading to unconscious movement loss. In another study, researchers found that the left thalamus was significantly larger than the right thalamus in patients with RRMS [19]. Specifically, they found that among patients with RRMS but without cognitive impairment, atrophy was observed exclusively in the left thalamus, whereas those with cognitive impairment displayed bilateral atrophy. Previous research has also shown that for individuals with few cognitive and physical impairments, brain atrophy can initially occur in one hemisphere and subsequently spread to the other as the disease progresses [19]. However, our study conducted a comprehensive analysis of the asymmetry index of subcortical structures in MS patients and identified only the globus pallidus structure as exhibiting statistically significant asymmetry. Thus, our study was based on prior research showing that asymmetry could be present across several subcortical structures in patients with MS and provided a thorough examination of the asymmetry indexes of subcortical structures.

Limitations

Our study had some limitations. An important limitation was the relatively small sample size, which could potentially weaken the statistical validity of the findings. The strict exclusion criteria in the study, which disqualified subjects with any cranial pathology or demyelinating manifestations, as well as neoplastic, degenerative, or vascular conditions that could be confused with MS, both reduced the sample size and limited the factors to be compared. Another limitation was that the participants' information was obtained from the hospital registration system, and cognitive assessment could not be performed on the participants due to the retrospective nature of the study. Finally, the study included patients with a diagnosis of RRMS in the patient group and those without any cranial pathology in the control group; if there was a disease outside the hospital records, this may affect the results.

## Conclusions

There is a need for a universally accepted measurement standard for subcortical structures, and it is essential to recognize and account for variability within each structure. The repeatability in subcortical structure volumes and volBrain measurements may accelerate the interest in studies of the human brain. Our study's results could serve as a crucial foundation and measure for assessing subcortical atrophy and asymmetry in MS, as well as for determining the disease's prognosis and the causes of its clinical symptoms. Subsequent research may profit from adopting our novel approach of considering brain atrophy as an end result rather than a predictor, which would enable a clearer understanding of the complex biological mechanisms that cause volume loss. In the future, volBrain may be used for analyzing, diagnosing, and treating MS. Future studies should consider investigating brain atrophy as an outcome rather than a predictor of MS, with the goal of elucidating the underlying biological mechanisms that contribute to volume loss.
